# Pain reduction with oral methotrexate in knee osteoarthritis, a pragmatic phase iii trial of treatment effectiveness (PROMOTE): study protocol for a randomized controlled trial

**DOI:** 10.1186/s13063-015-0602-8

**Published:** 2015-03-04

**Authors:** Sarah R Kingsbury, Puvan Tharmanathan, Nigel K Arden, Michael Batley, Fraser Birrell, Kim Cocks, Michael Doherty, Chris J Edwards, Toby Garrood, Andrew J Grainger, Michael Green, Catherine Hewitt, Rod Hughes, Robert Moots, Terence W O’Neill, Edward Roddy, David L Scott, Fiona E Watt, David J Torgerson, Philip G Conaghan

**Affiliations:** Leeds Institute of Rheumatic and Musculoskeletal Medicine and National Institute of Health Research (NIHR) Leeds Musculoskeletal Biomedical Research Unit, University of Leeds, Chapeltown Road, Leeds, LS7 4SA UK; York Trials Unit, Department of Health Sciences, Faculty of Science, Seebohm Rowntree Building, University of York, Heslington, York, YO10 5DD UK; NIHR Musculoskeletal Biomedical Research Unit, Nuffield Department of Orthopaedics, Rheumatology and Musculoskeletal Sciences, Nuffield Orthopaedic Centre, University of Oxford, Windmill Road, Headington, Oxford, OX3 7LD UK; Department of Rheumatology, Maidstone and Tunbridge Wells NHS Trust, Pembury, Tunbridge Wells, Kent TN2 4QJ UK; Musculoskeletal Research Group, Institute for Cellular Medicine, Newcastle University, Musculoskeletal Research Group, 4th Floor Catherine Cookson Building, The Medical School, Framlington Place, Newcastle University, Newcastle, NE2 4HH UK; Academic Rheumatology, Clinical Sciences Building, University of Nottingham, Nottingham City Hospital, Hucknall Road, Nottingham, NG5 1 PB UK; Department of Rheumatology and NIHR Wellcome Trust Clinical Research Facility, University Hospital Southampton NHS Foundation Trust, Southampton, SO16 6YD UK; Guy’s and St Thomas’ Hospitals Foundation NHS Trust, Great Maze Pond, London, SE1 9RT UK; The York Hospital, York Hospitals NHS Foundation Trust, Wigginton Road, York, YO31 8HE UK; Rheumatology Department, St Peters Hospital, Guildford Road, Chertsey, Surrey KT16 0PZ UK; Institute of Integrative Biology, University of Liverpool and Institute of Ageing and Chronic Disease, University Hospital Aintree, Longmoor Lane, Fazakerley, Liverpool, Merseyside L9 7AL UK; Arthritis Research UK Centre for Epidemiology, The University of Manchester, 2nd floor, Stopford Building, Oxford Road, Manchester, M13 9PT UK; Research Institute for Primary Care and Health Sciences, Keele University, Keele, Staffordshire ST5 5BG UK; Department of Rheumatology, King’s College London School of Medicine, King’s College London and Department of Rheumatology, King’s College Hospital, 10 Cutcombe Road, London SE5 9RJ, London, UK; Kennedy Institute of Rheumatology, Nuffield Department of Orthopaedics, Rheumatology and Musculoskeletal Sciences, University of Oxford, Windmill Road, Oxford, OX3 7HE, UK and Imperial College London, South Kensington Campus, London, SW7 2AZ UK

**Keywords:** Double-blind, Knee osteoarthritis, Methotrexate, Placebo-controlled, Randomized

## Abstract

**Background:**

Osteoarthritis (OA) is the fastest growing cause of disability worldwide. Current treatments for OA are severely limited and a large proportion of people with OA live in constant, debilitating pain. There is therefore an urgent need for novel treatments to reduce pain. Synovitis is highly prevalent in OA and is associated with pain. In inflammatory arthritides such as rheumatoid arthritis, methotrexate (MTX) is the gold standard treatment for synovitis and has a well-known, acceptable toxicity profile. We propose that using MTX to treat patients with symptomatic knee OA will be a practical and safe treatment to reduce synovitis and, consequently, pain.

**Methods/Design:**

Pain Reduction with Oral Methotrexate in knee Osteoarthritis, a pragmatic phase III trial of Treatment Effectiveness (PROMOTE) is an investigator-initiated, multi-centre, randomized, double-blind, pragmatic placebo-controlled trial. A total of 160 participants with symptomatic knee OA will be recruited across primary and secondary care sites in the United Kingdom and randomized on a 1:1 basis to active treatment or placebo, in addition to usual care, for 12 months. As is usual practice for MTX, dosing will be escalated over six weeks to 25 mg (or maximum tolerated dose) weekly for the remainder of the study. The primary endpoint is change in average knee pain during the past week (measured on an 11-point numerical rating scale) between baseline and six months. Secondary endpoints include other self-reported pain, function and quality-of-life measures. A health economics analysis will also be performed. A magnetic resonance imaging substudy will be conducted to provide an explanatory mechanism for associated symptom change by examining whether MTX reduces synovitis and whether this is related to symptom change. Linear and logistic regression will be used to compare changes between groups using univariable and multivariable modelling analyses. All analyses will be conducted on an intention-to-treat basis.

**Discussion:**

The PROMOTE trial is designed to examine whether MTX is an effective analgesic treatment for OA. The MRI substudy will address the relationship between synovitis and symptom change. This will potentially provide a much needed new treatment for knee OA.

**Trial registration:**

Current Controlled Trials identifier: ISRCTN77854383 (registered: 25 October 2013).

## Background

With a rapidly ageing population, osteoarthritis (OA) has become the fastest growing cause of disability worldwide [1,2]. Current estimates suggest that over 250 million people across the globe are affected by OA, with a lifetime risk for the development of knee OA of approximately 40% [1-3]. OA is characterized by chronic joint pain and functional impairment, resulting in markedly reduced quality of life for individuals. OA also places an enormous burden on health services and health economies, and is the second leading cause of absence from work [4]. The cumulative cost of OA has been estimated to amount to approximately 1% of gross national product [5,6].

One of the major barriers to reducing the impact of OA, both on individuals and society, is the lack of efficacious therapies available to treat the symptoms of OA or to slow the disease process and associated structural progression. Current management guidelines for OA include pharmacological therapies such as paracetamol and NSAIDs, and non-pharmacological therapies including weight loss and exercise [7-9]. Although current treatments are aimed at providing symptomatic relief, recent studies suggest that the large majority of people with OA live in constant pain despite use of available therapies [10]. These therapies are also associated with significant toxicities. In addition, because the typical OA patient is of advanced age with multiple comorbidities, many have contraindications to the use of traditional OA medications. Hence, there is a pressing need to identify alternative therapies for OA in order to tackle this increasing problem [11,12].

There is increasing evidence, particularly from imaging studies, of a high prevalence of synovitis (inflammation of the synovial membrane) in OA, with abnormalities present from the earliest stages of the disease and associated with the presence and severity of pain [13-18]. These changes are often indistinguishable to those observed in rheumatoid arthritis (RA), although they are generally confined to more discrete regions within the joint, generally adjacent to sites of chondropathy. Increased levels of pro-inflammatory cytokines (such as tumour necrosis factor (TNF)α, interleukin (IL)-1β and IL-6), reduced levels of anti-inflammatory cytokines (such as IL-10 and IL-1RA), infiltration of mononuclear cells and adaptive immune cell responses have all been demonstrated within OA fluid and tissue, suggesting that modulating the inflammatory response may be effective as a treatment target for OA [19-21].

The rationale for targeting synovitis as a treatment for OA pain is supported by previous studies of anti-inflammatory agents. Randomized controlled trials of non-steroidal anti-inflammatory drugs (NSAIDs) and the fewer randomized controlled trials of intra-articular (IA) and oral corticosteroids have demonstrated modest, short-term pain reduction associated with anti-inflammatory effects and reduced synovitis [22-27]. However, although NSAIDs and corticosteroids have positive short-term effects their long-term use is not desirable, with contra-indications to their use in many people with OA.

Disease modifying anti-rheumatic drugs (DMARDs) are the mainstay of treatment for RA and all have anti-synovial effects. Methotrexate ((MTX) 4-amino-10-methylfolic acid) is the first-line treatment widely used to treat synovitis in the inflammatory arthritides, and current evidence suggests it is the most effective DMARD and safe for long-term use [28]. Indications for the use of MTX have expanded in recent years to include trials to reduce cardiovascular disease (CVD) in the general population, as well as showing reductions in CVD in RA patients [29]. There is more uncertainty about the benefits of MTX in other peripheral joint arthritides such as psoriatic arthritis (PsA), however this may reflect a lack of robust trial data [30].

MTX is a folic acid antagonist which has both an anti-proliferative and an anti-inflammatory action. The anti-proliferative activity of MTX is mediated through inhibition of the *de novo* synthesis of purines and pyrimidines by acting as a specific antagonist of folic acid. MTX was initially used in high doses for its anti-proliferative effect in the treatment of cancer, at doses of up to 5,000 mg per week. At much lower doses (15 to 25 mg per week), as used in inflammatory arthritis, MTX has an anti-inflammatory effect by inducing an increase in adenosine release from cells through selective inhibition of aminoimidazole carboxamide ribonucleotide (AICAR) transformylase, an enzyme that catalyses an intermediary step in *de novo* purine biosynthesis. Extracellular adenosine is a potent inhibitor of inflammation, suppressing the inflammatory functions of neutrophils, macrophages and monocytes, dendritic cells and lymphocytes, thereby reducing secretion of inflammatory cytokines, including TNFα and IL-6, which drive synovitis [31,32].

Given the high levels of pro-inflammatory cytokines and the evidence of immune cell infiltration into OA joints, coupled with the strong correlations observed between synovitis and pain, there is rationale for the use of MTX for reducing symptoms in OA. Most patients tolerate MTX for long-term use [33] and the use of folic acid concomitantly with MTX reduces the incidence of side-effects [34]. Patients undergo regular blood monitoring to assess for toxicity, and abnormalities in results usually respond to a dose reduction or temporary cessation, or an increase in folic acid supplementation. Side-effects of MTX can include gastrointestinal side-effects, haematological abnormalities and elevated liver transaminases. Side-effects resulting in discontinuation of the drug vary in frequency from 15 to 17% [35,36], but have been shown to reduce to 4% in the second year of treatment [35].

**Figure 1:**
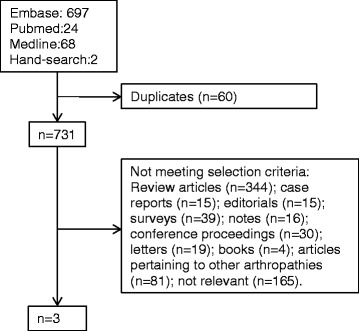
**Overview of systematic review of methotrexate use in osteoarthritis.** Databases: PubMed, MEDLINE and Embase. Search terms: MeSH headings #1 ‘osteoarthritis’ and #2 ‘methotrexate’. Limits: Humans.

To date there have been no large trials of MTX for treating OA. Two small studies, one in knee OA and the other in hand OA, have been carried out and these had conflicting results (Figure 1 and Table 1) [37,38]. One additional study examined the effect of MTX in calcium pyrophosphate crystal disease (CPPD) [39]. However, these studies included very low patient numbers and small doses of MTX, which makes it difficult to determine whether the results are valid.

**Table 1:** **Systematic review of methotrexate use in osteoarthritis**

**Reference**	**n**	**Site**	**Treatment**	**Outcome**
de Holanda 2007 [37]	58	Knee OA	Double-blind, placebo controlled, 4 months, 7.5 mg/week	No statistically significant difference between both groups regarding WOMAC (*P* = 0.94), Lequèsne Algofunctional Index (*P* = 0.87) and VAS (*P* = 0.89. No significant difference in paracetamol consumption between both groups, however, there was tendency to increased consumption in the placebo group.
Pavelka 2006 [38]	21	Erosive hand OA	Open label, 10 mg of MTX orally for two months	Significant decrease of pain after 2 months of treatment (54.4 ± 17.0 mm versus 39.7 ± 19.6 mm, *P* <0.01) and stiffness (28.8 ± 24 min versus 21.8 ± 19.1 min, *P* <0.01)
Chollet-Janin 2007 [39]	5	CPPD	Open label, 5 to 20 mg/week	Clinical response in all 5 patients with significant reduction in pain intensity, swollen and tender joint counts and mean improvement time of 7.4 weeks

CPPD, calcium pyrophosphate deposition disease; MTX, methotrexate; OA, osteoarthritis; VAS, visual analogue scale; WOMAC, Western Ontario and McMaster Osteoarthritis Index.

More recently, we conducted an open-label pilot study of 30 patients with knee OA who took MTX for six months [40]. A total of 23 patients completed the study, 20 of whom were taking a MTX dose of 15 mg/week or more. At six months, 50% of patients had a 20% reduction in pain, whilst 37% had a 40% reduction in pain. Of the seven participants who did not complete the study, four withdrew due to side effects (lethargy, nausea and headaches in three patients and thrush in one patient) and three withdrew due to lack of response. Further evidence for the potential of MTX as a therapy for OA can be extrapolated from animal studies, with positive effects on cartilage demonstrated in lapine models of OA [41,42].

Taken together these clinical and experimental studies suggest potential for MTX as a useful treatment for OA. We propose that treating patients with symptomatic knee OA with MTX will be a practical and safe treatment to reduce synovitis and therefore reduce pain. We believe that the preliminary data from the handful of small studies previously conducted, including our pilot study, strongly support the need for a well-designed, adequately powered, randomized placebo-controlled trial to examine fully the potential use of MTX as a treatment for OA. The Pain Reduction with Oral Methotrexate in knee Osteoarthritis, a pragmatic phase III trial of Treatment Effectiveness (PROMOTE) was designed to this end.

This paper states the objective of the PROMOTE trial, discusses study design challenges encountered in its planning, and outlines the resultant study design and protocol for the PROMOTE trial.

### Trial objectives

The primary objective of the trial is to determine whether MTX reduces pain associated with knee OA as compared to placebo. Secondary objectives of the trial are:

1. To determine whether MTX improves function,
2. To determine whether MTX improves quality of life and
3. To determine whether MTX is a cost-effective treatment for knee OA.

In addition to the main study, PROMOTE contains a magnetic resonance imaging (MRI) substudy and a biological substudy. Patients enrolled in the main study may opt to enrol in one, both or neither of the sub-studies. The objective of the MRI substudy is to provide an explanatory mechanism for associated symptom change by examining whether MTX reduces synovitis, and whether this is related to symptom change. The objective of the biological substudy is to determine whether baseline soluble immunological and inflammatory biomarkers predict response to treatment, and whether changes in levels occur following treatment with MTX.

### Trial development

The trial was designed with key stakeholders including rheumatologists with experience of treating OA, general practitioners with a special interest in musculoskeletal disease, methodologists and users with experience of knee OA. The intention was to be pragmatic, allowing physicians to include MTX in the overall care of patients with knee OA and have relative flexibility in this regard, thereby being representative of clinical practice. We faced a number of challenges in achieving the ideal design for the PROMOTE trial which are discussed in more detail in the relevant sections below. Key challenges included defining the dosing and dose escalation strategy for use of MTX in OA patients, maintaining the blind due to the well-known side-effect profile of MTX, timing of the primary outcome, ensuring optimal recruitment for a trial that is conducted in secondary care for a condition where patients predominantly reside in primary care and maintaining the pragmatic nature of the trial with respect to concomitant medication use, whilst protecting the validity of the primary pain outcome.

## Methods/Design

The PROMOTE trial is an investigator-initiated, multi-centre, 160 patient, randomized placebo-controlled trial to compare the reduction in pain associated with knee OA with MTX as compared to placebo. Participants will be randomized on a 1:1 basis to MTX or placebo, and treatment will be for 12 months.

### Population

All adults with symptomatic radiographic knee OA and inadequate response or toxicity to their existing medication (to include paracetamol, NSAIDs or opioid).

### Intervention and comparator

Participants will be randomized to receive either over-encapsulated MTX 2.5 mg tablets (packed with microcrystalline cellulose (Sharp Clinical Services (UK) Ltd, Crickhowell, Wales)) or placebo (matching capsules packed with microcrystalline cellulose (Sharp Clinical Services (UK) Ltd, Crickhowell, Wales)).

#### Choice of dosing for methotrexate in patients with osteoarthritis

MTX is currently used in RA with a maximum dose of 25 mg per week. Previous small studies in OA have used lower doses than those used in RA [37,38]; however to alleviate concerns around inefficacy due to inadequate dosing, we will follow current guidelines for use of MTX in RA as the model for the PROMOTE trial. Participants will be prescribed 10 mg MTX or placebo weekly for two weeks, followed by 15 mg weekly for two weeks, 20 mg weekly for two weeks and 25 mg weekly for the remainder of the study if there is no toxicity as determined at the physician’s discretion [43,44]. In practice, slower dose escalation may be necessary in some participants, and deviations from this protocol (for example escalation by 2.5 mg every two weeks) will be permitted at the clinician’s discretion to bring participants to the maximum tolerated dose as closely in line with the stated strategy as possible.

If participants show toxicity to MTX upon dose escalation the dose will be dropped to the maximum tolerated dose (minimum dose, 7.5 mg/week) and this will be maintained for the duration of the study. In our pilot study of MTX in OA, 20 of the 23 participants completing the study did so on a dose of 15 mg or more. Dose reduction (to 7.5 to 12.5 mg) was required in four participants; one due to renal impairment and three due to side effects (nausea, headache and sore roof of mouth), all of which improved upon dose reduction. Participants who are intolerant of oral MTX will not be switched to subcutaneous MTX (a common practice for RA), due to complexities in maintaining the blind.

All participants will be routinely reminded and encouraged to comply with the prescribed dose of investigational medicinal product (IMP). However, given the long half-life of MTX, those who miss doses of IMP will be allowed to restart medication if they so wish, and will not be handled differently during the course of follow-up. For the purposes of analysis, a participant missing more than four doses of MTX within any three-month period will be counted as a ‘non-complier’.

#### Non-investigational medicinal product

All participants will be prescribed oral folic acid 5 mg tablets to be taken on the six consecutive days after taking the weekly MTX or placebo dose. Folic acid supplementation has been shown to ameliorate side effects associated with MTX’s activity as an antagonist of folic acid metabolism [45].

#### Concomitant medication

In order to maintain the pragmatic nature of the trial, there are no restrictions written into the protocol with regard to concomitant analgesic medications. All participants, whether on MTX or placebo, will be allowed to continue taking the treatments for knee OA that they are taking at their screening visit for the duration of the trial. Investigators will be responsible for the overall management of a participant’s medication, and will ask participants to avoid changing their analgesic or anti-inflammatory medication for the duration of the trial. However, if a participant is experiencing increased pain and requires an increase in the dose of analgesics then the use of paracetamol, topical or oral NSAIDs or opioids, or a combination of these will be permitted, but the reason for the dose increase and the dose used will be documented. The choice of medications and doses to be used lie with the principal investigator and clinicians working at study sites in order to ensure that treatment for participants in both groups is optimized. Participants will be permitted to continue current use of chondroitin and glucosamine, provided the dose has been stable for three months at study entry; however their use must be clearly documented in the case report form (CRFChondroitin or glucosamine therapy will not be commenced during the duration of the trial. Chronic NSAID and opioid use days in the last three months) will be included as a covariate in the analysis.

The exception to this rule is corticosteroid use, since corticosteroids may have a significant effect on a participant’s experience of pain, to the point of affecting response to the primary outcome. Whilst there will be no overall restriction on the use of corticosteroids, the protocol provides guidance to investigators on the use of corticosteroids in order to minimise effects on the primary outcome, as detailed below. Participants will be asked not to use corticosteroids (oral, intravenous, IA or intra-muscular) between months three to six of the trial (three months before the primary endpoint). In months zero to three and six to 12 a single IA corticosteroid (in a non-knee joint) and up to one week of oral corticosteroids will be permitted. If corticosteroids are deemed necessary for medical reasons then their date of use, dose, route and indication must be clearly documented in the CRF. Patients will be offered the option of a rescue IA corticosteroid injection to their signal knee after the six-month visit of the trial if their symptoms are intolerable despite current medication. Corticosteroid use is ultimately at the discretion of the principal investigatoror study physician. All steroid use must be notified to the PROMOTE trial team centrally, but this will not affect the participant’s continued follow-up in the trial.

### Outcome measures

The PROMOTE trial will examine a range of clinical, imaging and quality-of-life and economic outcomes, in line with the objectives listed.

#### Clinical outcome measures

The primary endpoint of the study will be change in ‘average overall knee pain severity over the previous week’ (as graded on a zero to 10 numerical rating scale (NRS)) between baseline and six months (24 weeks). An NRS was chosen to measure pain as they have been found to be reliable and to demonstrate good face and criterion validity, and they are recommended as a core outcome measure for chronic pain clinical trials by the Initiative on Methods, Measurement, and Pain Assessment in Clinical Trials (IMMPACT) [46-50].

The timing of the primary outcome at six months was defined due to the slow-acting nature of MTX. Studies in RA have found that several months of treatment may be required before any symptomatic effect is noted [43,44]. Our own recent study of MTX versus MTX plus etanercept in RA, in which the MTX arm used the same dosing schedule as that planned for the PROMOTE trial, found that approximately 20% of participants in the MTX arm showed a major clinically significant response (tender and swollen joint count of zero) at three months, with this rising to 29% at six months and 33% at 12 months. In our pilot study of MTX in OA, the reduction in 48-hour pain VAS scores was significantly greater at 24 weeks (median (interquartile range (IQR)) reduction of 27 mm (four to 38)) than at 12 weeks (median (IQR) reduction of 9 mm (−1 to 36)).The dose escalation schedule for the PROMOTE trial requires a minimum of six weeks to reach maximum dosage, and in reality we know that it can often take longer than this to escalate the dosage. Taken together with evidence from the RA literature, which suggests that at least two to three months may be required at maximal dose for symptomatic benefit to be achieved, a primary outcome earlier than six months may fail to fully capture the clinical response to treatment. However, additional outcomes will be recorded at three months (12 weeks) to identify early response, and nine and 12 months (36 and 48 weeks) to capture longevity of response.

Secondary outcome measures include self-reported assessment of pain and function using 11-point NRSs to assess worst knee pain severity, global disease activity and satisfaction with knee function over the past week, together with the pain, function and stiffness subscales of the Western Ontario McMaster Universities Index (WOMAC version 3.1) and the Intermittent and Constant Osteoarthritis Pain scale (ICOAP). Depression and anxiety will be assessed using the Hospital Anxiety and Depression Scale (HADS). These measures are outlined further in Table 2.

**Table 2:** **Outcome measures**

	**Month(s)**				
	**0**	**3**	**6**	**9**	**12**
Primary outcome					
Average overall knee pain severity over the previous week (0 to 10 NRS)	✓	✓	✓	✓	✓
Secondary outcomes					
Imaging assessments:					
MRI of signal knee	✓		✓		
Clinical assessments:					
Knee examination	✓				
Self-reported questionnaires:					
WOMAC 3.1 (pain, stiffness and function) - five-point Likert scale	✓	✓	✓	✓	✓
ICOAP	✓	✓	✓	✓	✓
11-point NRS for:	✓	✓	✓	✓	✓
Worst knee pain severity,/ global disease activity and pain in other joints over the past week					
Satisfaction with knee function over the past 2 days					
Knee pain, aching or stiffness over the past month (no days to all days)					
Global^a^ improvement in knee problem, pain and function		✓	✓	✓	✓
Pain elsewhere (pain manikin)	✓	✓	✓	✓	✓
Duration of knee pain over the past 12 months (<7 days, 1 to 4 weeks, >1 month, <3 months or >3 months)	✓				
Onset of knee pain (last 12 months, 1 to 5 years, 5 to 10 years or 10 years or more)	✓				
Quality of life: SF-12 v2 and OAQoL [40]	✓		✓		✓
EuroQol EQ-5D [41,42]	✓		✓		✓
Depression and anxiety: HADS	✓		✓		✓
Resource use:	✓		✓		✓
Demographics and medical history	✓				
Brief medication questionnaire		✓	✓	✓	✓
Concomitant medication^b^	✓	✓	✓	✓	✓
Adverse events^b^		✓	✓	✓	✓

^a^A six-point Likert scale: completely better, much better, better, no change, worse or much worse. ^b^Also recorded at 1 and 2 months. HADS, Hospital Anxiety and Depression Scale; ICOAP, Intermittent and Constant Osteoarthritis Pain; NRS, numerical rating scale; OAQoL, Osteoarthritis Quality of Life Scale; VAS, visual analogue scale; WOMAC, Western Ontario McMaster Universities Index.

#### Quality-of-life and health economic outcome measures

Utility will be measured using the EuroQol (EQ-5D-5 L), deriving quality-adjusted life years (QALYs) for each participant. Disease-specific quality-of-life measures are listed in Table 2.

#### Imaging outcome measures

Gadolinium-enhanced 1.5/3.0 T MRI of the signal knee will be performed at baseline and six months according to the protocol outlined in Table 3. All images will be quality controlled by an experienced musculoskeletal radiologist within two weeks of acquisition to ensure that the image quality is sufficient for scoring purposes. MRIs will be centrally scored semi-quantitatively and quantitatively at the end of the study. Semi-quantitative scoring will be conducted using the MRI Osteoarthritis Knee Score (MOAKS) [51]. Images will be quantitatively scored using statistical shape modelling technology to automatically quantitate total and compartmental synovial volume, total and compartmental bone shape and other relevant features including cartilage morphology [52].

**Table 3:** **Magnetic resonance imaging acquisition protocol**

**Plane**	**Sequence**	**Slice Thickness**
Sagittal	T1 SE	2 mm
Sagittal	PD FSE TE = 40 Fat Saturated	3 mm
Sagittal	T2 FSE Fat Saturated	3 mm
Coronal	PD FSE TE = 40 Fat Saturated	3 mm
Coronal	STIR	4 mm
Axial	PD FSE TE = 40 Fat Saturated	3 mm
Post gadolinium:		
Sagittal	T1 3D SPGR Fat Saturated/Water Excitation	Isotropic

### Sample size

The standard deviation used in the sample size calculation is an estimate from previous studies. The internal pilot study will be used to re-estimate the sample size required and increase the recruitment target if necessary.

A data-driven analysis which related change on an NRS with patient global assessment of change using data from 10 similar trials of chronic pain showed that a 30% change, or two points on the NRS was related to change of clinical importance in chronic pain. The correlation between clinician and patient global assessment of change was high in this review, and the large sample size used underscores the external validity of these estimates [53]. This estimate of a two-point change on a NRS being associated with a ‘much better’ improvement was also seen in a prospective cohort of patients with chronic musculoskeletal pain [54].

In a recent placebo-controlled, 121 patient, OA knee trial using an 11-point pain NRS as the primary outcome, the mean (standard deviation (SD)) baseline pain score was 6.1 (2.1) in the control group, 7.7 (1.4) in the placebo group, 7.1 (2.8) in treatment group A and 6.7 (2.5) in treatment group B [55]. In a separate 73 patient OA knee trial, the mean (SD) baseline pain score was 4.0 (2.1) in group A and 4.3 (1.9) in group B. At 12 months, the mean (SD) within-group difference was 0.9 (2.1) for group A and 1.3 (2.4) for group B [56]. For the sample size calculation an SD of 4 is used as a conservative estimate.

The expected consistency in baseline pain among our trial population allows the use of raw change in score, rather than percentage change [53]. In order to detect a two-point change, with an assumed SD of 4 (80% power and 5% significance level), 64 participants per arm are required. Allowing for a conservative 20% dropout rate, a total of 160 participants will therefore need to be recruited into the study.

### Trial procedures

Ethical approval for the study has been granted by the Leeds West Research Ethics Committee (reference number 13/YH/0279). Informed written consent will be obtained from all participants.

### Participant recruitment strategy

Since knee OA is primarily managed within primary care, our recruitment strategy must enable a clear interface between primary care centres and the secondary care sites at which trial activity will take place. Relevant Clinical Commissioning Group (CCG) approvals will be obtained to enable primary care sites to act as Patient Identification Centres (PICs), with potentially eligible patients referred on to local secondary care study teams.

A total of 160 subjects with symptomatic knee OA will be recruited and randomly allocated to either the treatment or placebo control group. Recruitment methods will include advertisements through the local media and community groups, invitations to previous study participants who have given their consent to be contacted regarding future research projects and liaisons with musculoskeletal physicians, general practitioners and allied health professionals. Recruitment will be initiated at all sites as soon as local approvals are in place, therefore recruitment to the pilot phase may occur from multiple sites. There will be no pause in recruitment once 30 patients are recruited, as the outcome from the analysis of the pilot phase will be to either continue to the planned numbers or to increase the planned sample size.

### Informed consent and participant confidentiality

Informed consent will be obtained before patients are screened for participation in the PROMOTE trial. The right of the patient to refuse consent without giving reasons will be respected. Further, the patient will remain free to withdraw from the study at any time without giving reasons and without prejudicing any further treatment. The written consent will be obtained by an appropriately delegated clinician who is, by education and experience, qualified to do so, and who has signed and dated the staff authorization and delegation log. The process of obtaining written consent will be clearly documented in the patient’s medical notes. Patient confidentiality will be guaranteed at all times, in line with the requirements of the United Kingdom Data Protection Act 1998 and NHS regulations.

### Eligibility criteria

**Figure 2:**
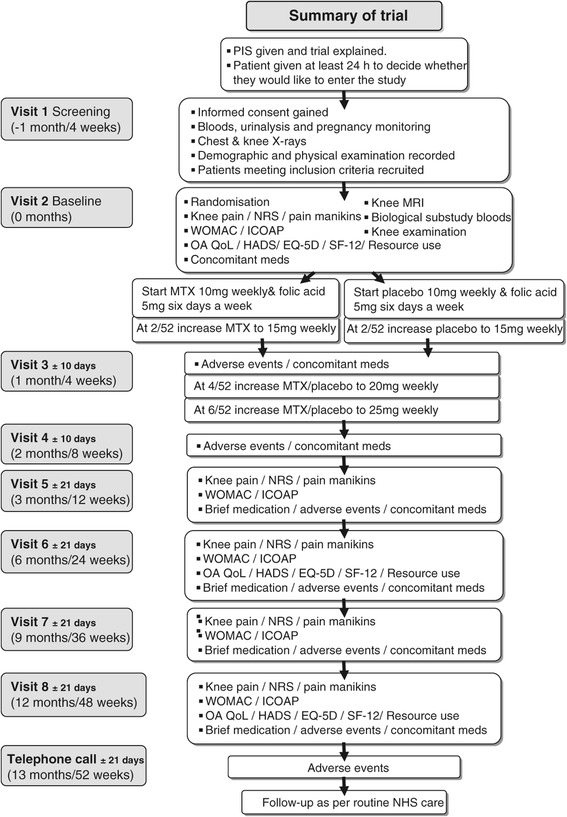
**Participant flowchart.** EQ-5D, EuroQol 5D-5 L; HADS, hospital anxiety and depression scale; ICOAP, intermittent and constant osteoarthritis pain scale; MRI, magnetic resonance imaging; MTX, methotrexate; NHS, National Health Service; NRS, numerical rating scale; OA QoL, osteoarthritis quality of life; PIS, patient information sheet; SF-12, Short Form-12; WOMAC, Western Ontario and McMaster Osteoarthritis Index. Treatment assignment and allocation concealment.

Participants must meet the inclusion and exclusion criteria (shown in Table 4) in order to participate. These will be assessed at the screening visit (Figure 2). Potential participants who are deemed ineligible at screening will be allowed a second screening visit if the reason for ineligibility is a temporary status (for example, recent corticosteroid injection; Table 4).

**Table 4:** **Eligibility criteria**

**Inclusion criteria**	**Exclusion criteria**
Fulfil clinical ACR Criteria for knee OA	The presence of any inflammatory arthritis (such as gout, reactive arthritis, rheumatoid arthritis, psoriatic arthritis, seronegative spondyloarthropathy, previous diagnosis of pseudogout in target joint with proven crystals on joint aspiration or elevated CRP at time of knee arthritis flare) or fibromyalgia.
Knee pain on most days in the last 3 months	
Knee pain is the predominant pain condition	Use of intra-articular (IA) hyaluronic acid in the signal knee within the 4 months preceding enrolment in the study^a^
Insufficient pain relief from, inability to tolerate or contra-indication to oral and/or topical NSAIDs and/or opioids. Moderate to severe pain of the signal knee as defined by a score of ≥40 mm on a VAS (0 to 100 mm) using the question ‘On average, how would you rate your knee pain during the last 3 months?’.	Use of IA, IM (intra-muscular) or oral corticosteroids in the 3 months preceding enrolment^a^
	Use of other anti-synovial agents (such as hydroxychloroquine or sulphasalazine) in the 2 months preceding the study^a^
	Significant knee injury or any knee surgery within the 6 months preceding enrolment in the study^a^
Patient able to identify a ‘signal’ painful knee (either the most painful knee or selected from equally painful knees)	The presence of non-OA causes of pain in the signal knee, such as referred hip pain, osteonecrosis or radicular spinal pain
A previous radiograph (X-ray) of the signal knee within the last 2 years with changes consistent with tibiofemoral OA	Commencement of physiotherapy or non-pharmacological knee OA treatment in the 2 months preceding the study^a^
No change in the average weekly dose of oral or topical analgesics (including NSAIDs) for at least 4 weeks^a^	A history of partial or complete joint replacement surgery in the signal knee at any time, listed for knee surgery or anticipating knee surgery during the study period
Has used chondroitin or glucosamine for at least 3 months with no change to the average weekly dose, is not using or is willing to stop using if recently started^a^	Women who are pregnant, breast-feeding or men or women planning pregnancy within 18 months after screening (approximately 6 months following last study medications)
All male and female subjects biologically capable of having children must agree to use a reliable method of contraception for the duration of the study and 24 weeks after the end of the study period. Acceptable methods of contraception are surgical sterilisation, oral, implantable or injectable hormonal methods, intrauterine devices or barrier contraceptives.	Use of any investigational (unlicensed) drug within 1 month prior to screening or within 5 half-lives of the investigational agent, whichever is longer^a^
	Have current signs or symptoms of severe, progressive or uncontrolled renal, hepatic, haematological, gastrointestinal, endocrine, pulmonary, cardiac, neurologic or cerebral disease
If female have potential for child bearing then a negative pregnancy test must be performed prior to starting treatment.	Poor tolerability of venepuncture or lack of adequate venous access for required blood sampling during the study period
The patient must be able to adhere to the study visit schedule and other protocol requirements	Uncontrolled disease states, such as moderate or severe asthma, COPD or inflammatory bowel disease, where flares are commonly treated with oral or parenteral corticosteroids, or recurrent infections
The patient must be capable of giving informed consent and the consent must be obtained prior to any screening procedures	Unwilling to keep alcohol intake to below the recommended maximum daily limit during the trial (2 units per day for women, 3 units per day for men)
All patients must have had a chest radiograph (X-ray) within the last 6 months	Planned need for live vaccination during 12 months of study (for example for foreign travel) with exception of Zostavax®, which is permissible
Aged ≥18 years	Melanoma or non-skin cancer in the past 3 years^a^
	Intolerance to lactose
	Significant haematological or biochemical abnormality:
	Haemoglobin ≤8.5 g/dL
	WCC ≤3.5 × 10^9^/L
	Neutrophils ≤1.5 × 10^9^/L
	Platelets ≤100 × 10^9^/L
	ALT >2 times ULN for the laboratory conducting the test.
	Creatinine >1.5 times ULN for the laboratory conducting the tested
	eGFR <30 mL/minute

^a^Criteria for which participants may be rescreened if they are ineligible at the initial screening visit. ALT, alanine aminotransferase; COPD, chronic obstructive pulmonary disease; eGFR, estimated glomerular filtration rate; IA, intra-articular; IM, intramuscular; IV, intravenous; RA, rheumatoid arthritis; ULN, upper limit of normal; VAS, visual analogue scale; WCC, white cell count.

Identical over-encapsulated MTX and placebo capsules will be produced to ensure allocation concealment. Upon production, study medication will be packed into bulk numbered bottles according to a randomization schedule. This will be prepared by the contract manufacturer using a computerized random number generator, thereby guaranteeing full allocation concealment. Study medication will be issued in numerical order by the pharmacy. Participants in each site will be randomly assigned to the intervention arm or the placebo arm in a ratio of 1:1, and the randomization will be double-blind.

### Blinding

Investigators and participants will remain blinded throughout the trial. Emergency unblinding will be allowed in limited situations that impact on the safety of study participants. Code-break envelopes for the full randomization schedule will be maintained by the Leeds General Infirmary pharmacy, and pharmacies at other sites will hold code-break envelopes for their respective participants. A 24-hour code-break telephone line will also be established in case of the need for emergency out-of-hours unblinding. Participants who are unblinded will be withdrawn from treatment but will continue to be followed as per the planned follow-up schedule, unless they request to fully withdraw from the trial.

#### Maintaining the treatment blind

Given the possible toxicities related to MTX, there is potential for investigators reviewing blood results to become aware of treatment. Therefore, patient-reported outcomes will be completed before any changes in dose or results of blood monitoring are discussed with participants. Where possible, members of staff with knowledge of blood results will not be responsible for administering patient questionnaires.

### Data collection

All data will be collected on standardized CRFs, which will be completed by site staff, verified by the principal investigator, and returned to the clinical trials unit for data entry. Study sites will also return a patient non-identifiable participant log and study drug dispensing log to the clinical trials unit.

#### Patient reported, clinical and imaging assessments

In addition to the patient-reported measures of pain, function and quality-of-life and imaging outcome measures described above and outlined in Table 2, a number of additional data elements will be collected during the trial. At the baseline visit, medical history and demographic data, including smoking and alcohol consumption, employment and family history of knee OA will be collected. Physical examination and vital signs, including height and weight, will be collected at screening, six and 12 months. A clinical examination of the knee will also be conducted at the baseline visit. This will assess effusion on a zero to three scale, where one is positive bulge sign, two is positive fluctuance (balloon sign) and three is tense effusion, and compartmental (medial tibiofemoral (MTF), lateral tibiofemoral (LTF) or patellofemoral (PF)) tenderness, also measured on a zero to three scale. The specific tools used to capture each data element are further detailed in Table 2.

#### Safety assessments

Adverse events will be recorded throughout the study. Intensity and relationship to the study medication will be ascribed.

#### Blood and urine safety assessment

Safety of therapy will be assessed according to regional monitoring guidelines and will be determined by treatment strategy. Full blood count (FBC), liver function test (LFT) and urea and electrolytes (U&E) tests will be performed at screening, every two weeks for the first eight weeks, and every four weeks thereafter. If a patient’s results remain abnormal despite action (such as reducing their MTX dose), or at clinician discretion, then study medication will be stopped. For the purposes of this study, abnormal is defined as: haemoglobin ≤8.5 g/dL; white cell count (WCC) ≤3.5 × 10^9^/L; neutrophils ≤1.5 × 10^9^/L; platelets ≤100 × 10^9^/L; alanine aminotransferase (ALT) > two times the upper limit of normal (ULN) for the laboratory conducting the test; creatinine >1.5 times the ULN for the laboratory conducting the test and estimated glomerular filtration rate (eGFR) <30 mL/minute.

C-reactive protein (CRP), anti-cyclic citrullinated protein (anti-CCP) and rheumatoid factor (RF) tests will be performed at screening. Anti-CCP and RF results from the previous 12 months may be used if these are available. A dipstick urinalysis will be performed at screening to check for evidence of leucocytes, blood or protein, to exclude urine infection. A urine dipstick pregnancy test will be performed for female participants with child-bearing potential, and appropriate contraceptive methods documented for those at risk.

### Data integrity and management

All data obtained will be kept strictly confidential and will be stored electronically on a database with secured and restricted access. Datasets for each subject will be identified by the participant trial identification number only.

### Withdrawal

All participants have the right to withdraw consent at any time without prejudice. At the time of withdrawal of consent, a full efficacy and safety evaluation should be performed if the participant consents. Participants who withdraw will be asked to complete the questionnaires as per the next planned study visit. At a participant’s request, their data collected up to the point of withdrawal can also be withdrawn from the trial and will not be used in the final analysis. Participants who withdraw will return to routine care in the rheumatology and musculoskeletal out-patient clinics.

In the event that a patient is unable to tolerate 7.5 mg of MTX, they will be withdrawn from treatment but will continue to be followed up on as per the planned follow-up schedule, unless they request to fully withdraw from the trial.

### Study site staff training

A centralized introduction and training session was held for all principal investigators and site staff. In addition to this, a site initiation visit will be held at all sites, in order to provide specific training to all staff involved in the study ahead of recruitment. Pre-study training will be conducted to ensure robustness of MRI acquisition.

### Trial site monitoring

The trial will be overseen and monitored by the York Trials Unit on behalf of the Sponsor, the University of Leeds. Each site will be assessed prior to site setup, and visited again once the third participant is recruited or at 20 weeks after the start of recruitment at site, whichever is sooner. The PROMOTE trial was assessed as low risk by the Medicines and Healthcare Products Regulatory Agency (MHRA), and therefore in addition to the single monitoring visit, procedures for central monitoring at the clinical trials unit have been put in place. This will mainly involve cross-checking logs returned from the research team and site pharmacy for consistency. A data monitoring and ethics committee will provide independent oversight to ensure data quality and compliance with the trial protocol.

### Statistical analysis

Full details of the statistical methods will be written in an approved statistical analysis plan prior to any data analysis.

### Primary endpoint analysis

The primary analysis will be conducted on the intention-to-treat population, including all randomized participants in the groups to which they were randomized. A per protocol population (excluding major protocol violations) will be used to check the robustness of the primary analyses. The safety population will consist of all patients receiving at least one dose of the study drug.

The first 30 participants (included in the internal pilot study) will be included in the main trial analysis along with the rest of the participants. Wittes and Brittain show that sample size re-estimation using an internal pilot study has minimum impact on the overall type I error, therefore an alpha level of 0.05 will still be used for the primary analysis [57].

The pilot phase analysis, which will be conducted after primary outcome data becomes available for the thirtieth participant, will include the following:

1. Assessment of the variability of the primary outcome measure, the estimate (SD) will be used to update the sample size calculation;
2. Consent rate (out of the number of information packs sent out during the pilot phase);
3. Retention rates associated with each treatment and
4. Return rate for the six-month outcome assessment.

The primary analysis for the main study will be conducted using linear regression adjusting for the baseline measure. Treatment groups will be compared at six months with respect to their average overall knee pain severity over the previous week.

All secondary analyses will be conducted using linear or logistic regression, depending on the type of outcome measure, adjusting for the same covariates as the primary analysis. The intention-to-treat population will be used for all secondary analyses. All secondary outcomes will be described descriptively (mean, SD, median, minimum and maximum for continuous data and counts and percentages for categorical data). The Short Form-12 questionnaire will be summarized for all components. To minimise multiple testing, only the overall physical component score and mental component score will be analysed, using the same analysis methods as for the primary outcome. For continuous outcomes the regression model assumptions will be checked and, if necessary, data will be transformed prior to analysis if this improves the model fit, or normalises the distribution of residuals.

Adverse events will be coded according to the MedDRA adverse event dictionary. The overall incidence of serious adverse events and adverse events and number and proportion of patients reporting such events will be summarized by treatment group. Adverse events (including serious adverse events) will be summarized by the number and percentage of subjects who experienced the event by system organ class and preferred term. Subjects will only be counted once. If a subject reports the same adverse event more than once then the maximum grade and strongest causal relationship to study treatment will be used for the summary tables. Adverse events will also be summarized by severity and by relationship to study drug. All adverse events will be included in individual subject line listings.

Analysis of MRI endpoints will be exploratory. Treatment groups will be compared with respect to changes in synovitis, cartilage and bone using linear regression models with adjustment for the same covariates as the primary analysis. Linear regression models will also be used to explore the association between symptom outcome and baseline MRI measurements. Changes in MR features and the relationship of these changes with symptom changes will be explored descriptively.

### Health economics analysis

An economic analysis will be undertaken in order to determine the cost-effectiveness of oral MTX for the treatment of knee OA. A cost-utility analysis will also be undertaken to explore the impact on health-related quality of life. The analyses will be conducted from the perspective of the United Kingdom NHS and Personal Social Services (PSS), in line with National Institute for Health and Care Excellence recommendations [58].

Health benefits for the economic evaluation will be measured in terms of pain reduction and QALYs. The QALY is a generic outcome which enables decision makers to compare across different disease areas. QALYs will be generated from the EuroQol EQ-5D-5 L for use in the cost-utility analysis, and will be estimated by measuring the area under the curve [59]. Utility data will be collected at the same time points as for clinical outcomes (at baseline, six months and 12 months).

Costs of the intervention, control and the total health care costs during the treatment and follow-up period will be assessed, including costs of adverse events and medications. Health service resource use will be collected via a self-reported resource use questionnaire, with national costs applied to the quantities of resources utilized, for example using NHS Reference Costs [60] and Unit Costs of Health and Social Care [61]. The unit costs of medications will be sourced from the British National Formulary [62].

The within-trial analysis will use regression methods to allow for the correlation between cost and outcome data generated from the trial, with adjustment for covariates. A cost-effectiveness analysis will determine the cost per unit of reduction in knee pain score (as measured on the NRS). A cost-utility analysis will be undertaken to generate the cost per QALY. The results will be presented in terms of incremental cost-effectiveness ratios (ICERs). Future costs and outcomes will not be discounted due to the trial follow-up being one year. Cost-effectiveness acceptability curves will be produced to explore the probability that MTX will be cost-effective at different cost-effectiveness thresholds [63].

## Discussion

Current medical therapy does not provide satisfactory pain relief in the majority of people with OA [64]; with the rapidly ageing population the prevalence of OA will continue to rise over the next decade. Unless the barriers to successful treatment of OA are overcome, the already considerable social and economic burden will soon reach an unsustainable level. The last decade has seen major advances in understanding OA pathology. MRI has demonstrated that synovial inflammation is common in OA and is related to symptoms. MTX is the most common agent used to treat synovitis in RA and because of its favourable efficacy, long-term retention rates, low cost and established track record it has become the standard by which other DMARDs are evaluated, supporting our choice to use MTX rather than any other DMARD for a trial in OA. Since the design of this study and conduct of the systematic literature review, a single-centre randomized controlled trial of MTX in knee OA has been published [65]. The study demonstrated a clinically relevant reduction in knee pain and physical function in the intervention group compared with the placebo group at 28 weeks. A significantly higher proportion of patients in the MTX group (53%) showed a reduction in VAS of more than 20 mm compared to the placebo group (24%; *P* = 0.018). The positive findings of this study reinforce the potential of MTX as a treatment for OA. However, given the substantial impact that using MTX for treating OA would have on clinical practice, a large multi-centre confirmatory study is essential. The PROMOTE trial has been designed to be pragmatic in nature so as to answer the question as to whether MTX is a valuable addition to the overall management of patients with OA.

## Trial status

Recruitment to the trial began in June 2014. Follow-up of participants is in progress and is expected to be completed in December 2016.

## Abbreviations

AICAR: Aminoimidazole carboxamide ribonucleotide
ALT: Alanine aminotransferase
CCG: Clinical Commissioning Group
CCP: Cyclic citrullinated protein
CCRN: Comprehensive Clinical Research Network
CPPD: Calcium pyrophosphate crystal disease
CRF: Case report form
CRP: C-reactive protein
CVD: Cardiovascular disease
DMARD: Disease modifying anti-rheumatic drug
eGFR: Estimated glomerular filtration rate
FBC: Full blood count
GP: General practitioner
HADS: Hospital Anxiety and Depression Scale
IA: Intra-articular
ICER: Incremental cost-effectiveness ratio
ICOAP: Intermittent and Constant Osteoarthritis Pain
IL: Interleukin
LFT: Liver function test
LTF: Lateral tibiofemoral
MHRA: Medicines and Healthcare Products Regulatory Agency
MOAKS: MRI Osteoarthritis Knee Score
MRI: Magnetic resonance imaging
MTF: Medial tibiofemoral
MTX: Methotrexate
NHS: National Health Service
NICE: National Institute for Health and Care Excellence
NIMP: Non-Investigational Medicinal Product
NRS: Numerical rating scale
NSAID: Non-steroidal anti-inflammatory drug
OA: Osteoarthritis
OA QoL: Osteoarthritis Quality of Life Scale
PF: Patellofemoral
PICS: Patient Identification Centres
PsA: Psoriatic arthritis
PSS: Personal Social Services
QALY: Quality-adjusted life year
RA: Rheumatoid arthritis
RF: Rheumatoid factor
SD: Standard deviation
TNF: Tumour necrosis factor
U&E: Urea and electrolytes
ULN: Upper limit of normal
VAS: Visual analogue scale
WCC: White cell count
WOMAC: Western Ontario and McMaster Universities Index of OA
